# Serum CA19‐9, CA‐125 and CEA as tumor markers for mucinous ovarian tumors

**DOI:** 10.1111/jog.14427

**Published:** 2020-08-23

**Authors:** Arb‐aroon Lertkhachonsuk, Supree Buranawongtrakoon, Navamol Lekskul, Naparat Rermluk, Wei‐Wei Wee‐Stekly, Chuenkamon Charakorn

**Affiliations:** ^1^ Division of Gynaecologic Oncology, Department of Obstetrics & Gynaecology, Faculty of Medicine Ramathibodi Hospital Mahidol University Bangkok Thailand; ^2^ Department of Pathology, Faculty of Medicine Ramathibodi Hospital Mahidol University Bangkok Thailand; ^3^ Minimally Invasive Surgery Unit, Department of Obstetrics and Gynecology KK Women's and Children's Hospital Singapore

**Keywords:** CA‐125 antigen, CA19‐9 antigen, carcinoembryonic antigen, ovarian cancer, ovarian neoplasm

## Abstract

Abstract

**Aim:**

To analyze the use of serum cancer antigen 19‐9 (CA19‐9), cancer antigen 125 (CA‐125) and carcinoembryogenic antigen (CEA) in predicting the malignant potential of mucinous ovarian tumor, and to assess the clinical factors associated with these tumors.

**Methods:**

This retrospective study collected the data from 314 patients who were diagnosed with mucinous ovarian tumor. These patients had preoperative serum CA19‐9, CA‐125, CEA available and underwent surgery at Ramathibodi Hospital between January 2010 and December 2016. The diagnostic performance of CA19‐9, CA‐125 and CEA was analyzed using the receiver operator characteristic curve. The associations between clinicopathological factors and serum CA19‐9, CA‐125 and CEA level were also analyzed.

**Results:**

A total of 314 patients were recruited in this study. They consisted of 221 patients with benign mucinous ovarian tumor (70.38%), 65 patients with borderline mucinous ovarian tumor (20.70%) and 28 patients with mucinous ovarian carcinoma (8.92%). Multivariate analysis revealed that the tumor size, elevated serum CA19‐9, CA‐125 and CEA influenced the tumor pathology. The mucinous ovarian tumor with large tumor size, elevated serum CA19‐9, CA‐125 and CEA more than the cut off values showed a positive correlation with the risk ratio of 1.60 (95% CI = 1.13–2.28; *P* = 0.005), 1.74 (95% CI = 1.22–2.47; *P* = 0.002), 1.90 (95% CI = 1.34–2.70; *P* < 0.001), 1.58 (95% CI = 1.10–2.29; *P* = 0.020), respectively. CA‐125 provided the highest diagnostic performance, with an area under receiver operator characteristic curve of 0.745, to differentiate between borderline, malignant or benign mucinous ovarian tumor.

**Conclusion:**

Preoperative elevation of the serum CA19‐9, CA‐125, CEA and tumor size are useful predictors to differentiate between benign, borderline and malignant mucinous ovarian tumor. The best predictor is CA‐125, followed by CA19‐9 and CEA.

## Introduction

Mucinous ovarian tumors account for 15% of epithelial ovarian tumors.^1^ These can be categorized according to the histopathologic characteristics and clinical behaviors, as benign mucinous ovarian tumors, mucinous borderline ovarian tumors (mBOT) and mucinous ovarian carcinoma. Mucinous ovarian tumor usually presents with a huge pelvic mass, with or without complication(s), symptomatic or asymptomatic at the diagnostic time. The ultrasonographic features can be either a huge uniloculated or multiloculated ovarian mass. If there are solid nodules, or presence of ascites fluid, borderline or malignant ovarian tumors are to be suspected.

Management of mucinous ovarian tumors is not different from other histopathologic cell types of ovarian tumor. Factors to be considered include age, fertility‐desire, unilateral or bilateral involvement, chances of mBOT or malignancy, patients' health conditions and surgeons' experiences. In the benign condition, if the patients are young and desire fertility, the unilateral salpingooophorectomy or ovarian cystectomy is the treatment of choice depending on the intraoperative findings. If the bilaterality is present, unilateral salpingooophorectomy with unilateral ovarian cystectomy, or bilateral ovarian cystectomy can be considered. If mBOT is suspected, the surgery adopted can be similar to that as for a benign case; however the excision of suspected peritoneal lesions, omentectomy or appendectomy if the appendix appears abnormal should also be performed. The surgery for a malignant mucinous ovarian tumor is the same as other epithelial ovarian malignancies; which can be fertility‐sparing for stage I. The roles of intraoperative frozen section for mucinous ovarian tumors are limited. The accuracy of the frozen section when compared to the final pathological diagnosis has been reported at 65–70% for benign and mBOT because of the large diameter and high heterogeneity. This may be due to the enormous size of mucinous tumors that require sampling.^2^, ^3^ Preoperative counseling for ovarian tumors suspicious for malignancy is very important. As a result of the similar clinical presentations or ultrasonographic findings of mucinous tumors, the roles of tumor markers for the discrimination of the tumors are still controversial.

Cancer antigen 19‐9 (CA19‐9) is elevated mostly in gastrointestinal, biliary tract or ovarian tumors, particularly in mucinous type.^1^, ^4^, ^5^, ^6^, ^7^, ^8^ Santotoribio and colleagues reported the sensitivity and specificity of CA19‐9 in ovarian carcinoma screening were 50% and 97.6%, respectively.^9^ Other studies showed controversial results, and a limited number of them focused on the association between mucinous ovarian tumor and CA19‐9.^1^, ^10^ Cancer antigen 125 (CA‐125) is utilized in mucinous ovarian tumors to predict the risk of malignancy by calculating risk of malignancy index score which is extensively studied. Approximately 85% of patients with epithelial ovarian cancer had CA‐125 levels of greater than 35 U/mL.^11^ Carcinoembryogenic antigen (CEA) rises in colonic and ovarian carcinoma, with the normal value of <5 ng/mL.^8^, ^12^ Therefore, CA19‐9, CA‐125 and CEA appeared to be important in gynecologic malignancy screening. This research aimed to evaluate the performance of these tumor markers in differentiating between benign, and borderline or malignant mucinous ovarian tumors.

## Methods

After approval from the Ramathibodi Institutional Review Board, this retrospective study collected the data from 314 patients who presented with ovarian mass (es). They were admitted for surgery under the Obstetrics and Gynecology Department, Faculty of Medicine Ramathibodi Hospital, Mahidol University between January 2010 and December 2016. The inclusion criteria included those who were diagnosed with mucinous ovarian tumor by pathological confirmation with available information of preoperative serum CA19‐9, CA‐125 and CEA. The exclusion criteria included those with incomplete medical records and those who were diagnosed with other malignancies. All the pathological slides were reviewed by a gynecologic pathologist to confirm the final diagnosis based on 2014 WHO classification and rule out metastatic mucinous tumor. The demographic data included age, parity, menopausal status, intraoperative tumor size, tumors' complication (i.e., rupture, leakage, or torsion), pathological results (i.e., benign, borderline or malignant) and serum tumor marker before the surgery (CA19‐9, CA‐125 and CEA with cut off values at 39 U/mL, 35 U/mL and 3.8 ng/mL, respectively). Descriptive statistics were summarized by frequency and percentage, mean and standard deviation (SD), or median and interquartile range, according to the distribution of data. The univariate analysis was applied by Pearson's chi‐squared or Fisher's exact test for categorical data and Student *t*‐test or Wilcoxon rank‐sum (Mann–Whitney *U*) test for continuous data, depending on the distribution of the data. A *P*‐value of 0.10 was used as a cutoff threshold to select variables for multivariate analyses. Taken into account of the outcome of interest, which was the borderline or malignant pathological results, multiple logistic regression analysis was performed by forward selection method. A *P*‐value of 0.05 was considered as the level of statistical significance. The diagnostic performance of these tumor markers was obtained by the receiver operating characteristic (ROC) curve, from which the area under the curve was used to compare each tumor marker in the discrimination between benign, borderline or malignant mucinous ovarian tumor. All analyses were performed using stata version 14.2 statistical software (StataCorp).

## Results

The data of mucinous ovarian tumor patients who underwent surgery and preoperative examination for serum CA‐19‐9, CA‐125, and CEA at Ramathidodi Hospital during January 2010 until December 2016 revealed 314 records. There were 221 patients with benign mucinous ovarian tumor (70.38%), 65 patients with borderline mucinous ovarian tumor (20.70%) and 28 patients with mucinous ovarian carcinoma (8.92%). The mean age was 47.3 years old. The majority of the patients were multiparous (61.5%) and in premenopausal status (51.13%). There were 220 patients (72.1%) with serum CA19‐9 ≤ 39 U/mL, 212 patients (67.7%) with serum CA‐125 ≤ 35 U/mL and 250 patients (83.9%) with serum CEA ≤ 3.8 U/mL. For the tumors' characteristic, the median of tumor size was 12 cm (range 2–60 cm). Twenty‐six patients presented with bilateral tumor (8.3%) and 22 patients (7.0%) had complications (torsion, rupture or leakage). The patients' and tumors' characteristics by pathological classification are demonstrated in Table 1. Univariate analysis revealed the serum CA19‐9, serum CA‐125, serum CEA, tumor size and tumor complications were factors for the multivariate analysis. Results of the multivariate analysis revealed that tumor size > 12 cm and elevated CA19‐9, CA‐125 and CEA influenced tumor pathology as demonstrated in Table 2. The mucinous ovarian tumor with tumor size > 12 cm, elevated CA19‐9, CA‐125 and CEA more than the cut off values showed the positive correlation with the risk ratio (RR) of 1.60 (95% CI = 1.13–2.28; *P* = 0.005), 1.74 (95% CI = 1.22–2.47; *P* = 0.002), 1.90 (95% CI = 1.34–2.70; *P* < 0.001), 1.58 (95% CI = 1.10–2.29, *P* = 0.020), respectively. The sensitivity and specificity of each tumor marker (CA19‐9, CA‐125 and CEA) in borderline or malignant mucinous ovarian tumor for CA19‐9 were 52.7% and 83.8%, CA‐125 were 68.2% and 83.9%, CEA were 31.9% and 90.8%, respectively.

**Table 1:** Clinical characteristics of patients with mucinous tumors

	Benign mucinous tumor	Borderline or malignant mucinous tumor	*P*‐value
Patient characteristics			
Age (year); mean (SD)	46.6 (14.6)	49.0 (14.6)	0.19
Parity; median (range)	1 (0–8)	1 (0–7)	0.54
Menopause			
No	113 (51.1)	47 (50.5)	0.92
Yes	108 (48.9)	46 (49.5)	
Serum CA19‐9			
≤39 U/mL	176 (83.8)	44 (47.3)	<0.001
>39 U/mL	34 (16.2)	49 (52.7)	
Serum CA‐125			
≤35 U/mL	173 (78.6)	39 (42.0)	<0.001
>35 U/mL	47 (21.4)	54 (58.1)	
Serum CEA			
≤3.8 U/mL	188 (90.8)	62 (68.1)	<0.001
>3.8 U/mL	19 (9.2)	29 (31.9)	
Tumor characteristics			
Tumor size (cm); median(range)	10 (2–60)	18 (3–40)	<0.001
Bilateral involvement			
No	203 (91.9)	85 (91.4)	0.89
Yes	18 (8.1)	8 (8.6)	
Torsion/rupture/leakage			
No	209 (94.6)	83 (89.3)	0.092
Yes	12 (5.4)	10 (10.8)	

Values are presented as number (%).

**Table 2:** Factors associated with borderline pathology or malignancy in mucinous ovarian tumor: multivariate analysis

Factors	Risk ratio	95% confidence interval	Standard error	*P*‐value
Tumor size (>12 cm)	1.60	1.13–2.28	0.301	*P* = 0.005
CA19‐9 > 39 U/mL	1.74	1.22–2.47	0.320	*P* = 0.002
CA12‐5 > 35 U/mL	1.90	1.34–2.70	0.308	*P* < 0.001
CEA > 3.8 U/mL	1.58	1.10–2.29	0.388	*P* = 0.020

**Figure 1:**
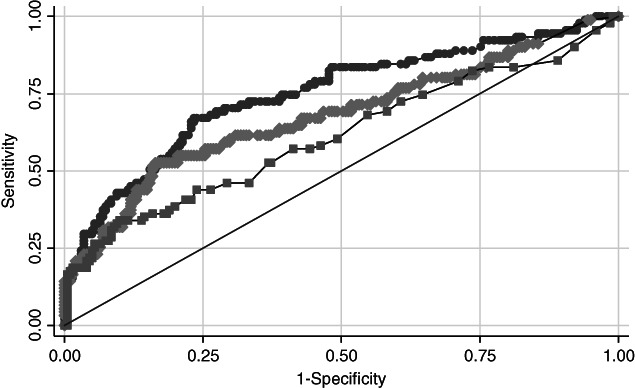
Diagnostic performance compared between CA19‐9, CA12‐5 and CEA for the discrimination of borderline or malignant and benign mucinous ovarian tumor. (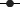) CA‐125 ROC area: 0.7473. (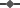) CA19‐9 ROC area: 0.6793 (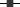) CEA ROC area: 0.6122. (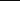) Reference

Table 3 demonstrated the diagnostic performance of each tumor marker in discrimination between borderline or malignant and benign mucinous ovarian tumor. Comparing between each tumor markers, the CA‐125 provided the highest diagnostic performance with area under ROC curve of 0.74 as shown in Figure 1.

**Table 3:** Diagnostic performance of each tumor markers for discrimination of borderline or malignant and benign mucinous ovarian tumor

Tumor markers	Sensitivity	Specificity	Area under ROC curve (95% CI)
CA19‐9	52.7%	83.8%	0.686 (0.616–0.756)
CA12‐5	68.2%	83.9%	0.745 (0.683–0.808)
CEA	31.9%	90.8%	0.610 (0.535–0.685)

## Discussion

In predicting malignancy in ovarian tumor patients, besides history taking and physical examination, the use of tumor markers as a part of evaluation is also important. In this study, we focused on mucinous ovarian tumor, which is a subtype of epithelial ovarian tumor. The well‐known tumor marker used in the preoperative evaluation of epithelial ovarian tumor is CA‐125^12^, ^13^ but there are limited data in the mucinous ovarian tumor group. There is evidence supporting stronger association between serum CA19‐9 and mucinous cell type than other cell types of epithelial ovarian tumors.^1^, ^5^ Serum CEA is also commonly used as a tumor marker in mucinous epithelial ovarian tumor.^12^ This study evaluated these 3 serum tumor markers (CA19‐9, CA‐125, and CEA) and established their diagnostic value in the prediction of malignancy in mucinous ovarian tumors. Also there has been published data that these tumor markers are also helpful even in improvement of the accuracy of the frozen section in mucinous ovarian tumors.^3^

This retrospective study collected data of 314 patients, which was a larger sample size than that in previous studies. In addition to these serum tumor markers, tumor size is also related to the association of the malignant or borderline pathological diagnosis of mucinous ovarian tumor in our findings. In our study, larger tumor size correlated with borderline or malignant tumors with the median of 18 cm (range 3–40 cm), while that in the benign mucinous tumor group was 10 cm (range 2–60 cm). This result was similar to the study by Cho H.Y. and colleagues published in 2014,^5^ which demonstrated the association between the serum CA19‐9 and the tumor size. Furthermore, they also reported that elevation of serum CA19‐9 was associated with the tumor pathology. In their study, the tumor size was determined by pre‐operative ultrasonography and used a cutoff of 15 cm. Similarly, Kelly P.J. and colleagues found that elevated serum CA19‐9 was associated with the tumor maximal dimension.^1^ In their study, the median of tumor dimension in the primary ovarian mucinous carcinoma group was 19 cm. In our study, we collected the data from the operative notes and calculated the mean tumor size according to the final pathologic reports which was different from the mentioned studies. However, the result for the tumor size was largely similar.

All of the three serum tumor markers (CA19‐9, CA‐125, and CEA) had strong association with the tumor pathology. The elevation above the widely used cut‐off levels of the serum CA19‐9 (>39 U/mL), CA‐125 (>35 U/mL) and CEA (>3.8 U/mL) was significantly associated with the borderline or malignant mucinous tumor with the risk ratio of 1.74 (95% CI = 1.22–2.47), 1.90 (95% CI = 1.34–2.70) and 1.58 (95% CI = 1.10–2.29), respectively. Moreover, the serum CA‐125 demonstrated the strongest association with the diagnosis of borderline or malignancy among those mucinous ovarian tumors, followed by CA19‐9 and CEA. The results correspond to the studies by Tamakoshi,^4^ Cho^5^ and Kelly.^1^ These serum tumor markers also demonstrated good diagnostic performance. The area under ROC curve of CA19‐9, CA‐125 and CEA was 0.68 (95% CI = 0.61–0.75), 0.74 (95% CI = 0.68–0.80), 0.61 (95% CI = 0.53–0.68) respectively. Among these markers, the serum CA‐125 had shown the best diagnostic performance, followed by CA19‐9 and CEA.

Our study is a study that evaluated the diagnostic performance of serum CA‐125, CA19‐9 and CEA in mucinous ovarian tumor. Although CA‐125 is the best serum marker in our results, CA19‐9 and CEA were comparable. The results not only answered our research questions, but also could be applied in clinical practice as one of the preoperative tools for the differential diagnosis between borderline or malignant and benign mucinous ovarian tumors. The limitations of this study were the retrospective nature of this research, selection and information bias and incomplete patient's data. In addition, routine preoperative evaluation of these serum tumor markers has yet to be implemented.

Preoperative elevation of the serum CA19‐9, CA‐125 and CEA was a useful predictor to differentiate between benign and borderline or malignant mucinous ovarian tumor. In our findings, serum CA‐125 had the best diagnostic performance, followed by serum CA19‐9 and CEA, respectively. Tumor size was also a significant associating factor. We suggest that the patients with mucinous ovarian tumor who were suspicious for borderline pathology or malignancy from the clinical manifestation, should undergo preoperative imaging for evaluation of the tumor size. In addition, these three serum tumor markers should also be preoperatively obtained in order to make a proper surgical plan.

## Disclosure

None declared.
